# Reciprocal relationships and the importance of feedback in patient and public involvement: A mixed methods study

**DOI:** 10.1111/hex.12684

**Published:** 2018-04-14

**Authors:** Elspeth Mathie, Helena Wythe, Diane Munday, Paul Millac, Graham Rhodes, Nick Roberts, Nigel Smeeton, Fiona Poland, Julia Jones

**Affiliations:** ^1^ CRIPACC University of Hertfordshire Hatfield UK; ^2^ Public Involvement in Research Group CRIPACC University of Hertfordshire Hatfield UK; ^3^ INsPIRE Patient and Public Involvement in Research Bedfordshire and Peterborough UK; ^4^ School of Health Science University of East Anglia Norwich UK

**Keywords:** feedback, health, patient and public involvement, public, research

## Abstract

**Background:**

Reciprocal relationships between researchers and patient and public involvement (PPI) contributors can enable successful PPI in research. However, research and anecdotal evidence suggest that researchers do not commonly provide feedback to PPI contributors thus preventing them from knowing whether, how or where their contributions were useful to researchers and research overall.

**Aims:**

The aim of this study was to explore the variation, types, importance of, and satisfaction with feedback given by researchers to PPI contributors in six PPI groups in England, and identify the barriers to the process of feedback.

**Methods:**

An explanatory mixed methods sequential study design with a questionnaire survey followed by semi‐structured interviews with researchers and PPI contributors in six PPI groups. PPI contributors were involved in all stages of the research process.

**Results:**

Researchers do not routinely give feedback to PPI contributors. Feedback was found to have different meanings: an acknowledgement, impact and study success and progress. PPI contributors who receive feedback are motivated for further involvement; it supports their learning and development and prompts researchers to reflect on PPI impact. The importance of the role of a PPI lead or coordinator to facilitate the process of providing feedback was also highlighted.

**Conclusion:**

This study found no generic way to give feedback indicating that mutual feedback expectations should be discussed at the outset. PPI feedback needs to become integral to the research process with appropriate time and resources allocated. PPI feedback can be seen as a key indicator of mature, embedded PPI in research.

## 1 INTRODUCTION

Valuing and strengthening relationships between researchers and patient and public contributors have been identified as a key component in enabling successful, embedded Patient and Public Involvement (PPI) in research.^1^ Good communication, reciprocal trust and redressing unequal power relationships between researchers and members of the public have been identified in systematic reviews and literature as key elements in Patient and Public Involvement.^2^, ^3^, ^4^, ^5^, ^6^, ^7^ Different models of working together include consultation, collaboration and, increasingly, co‐production.^8^, ^9^, ^10^, ^11^ Providing feedback to PPI contributors regarding the usefulness of their contributions can be viewed as an important way of developing positive relationships between researchers and PPI contributors. However, research as well as anecdotal evidence suggests that routine feedback rarely occurs.^1^, ^2^, ^12^, ^13^

Researchers were first reported as failing to feedback to PPI contributors 10 years ago in the United Kingdom and was noted as “the single biggest issue raised by volunteers”^12^, ^14^ ^(p.65)^. More recently, a qualitative systematic review by Baylis et al^15^ reported that PPI contributors wanted detailed feedback on how their comments had influenced the final review paper and on their need for further training. When feedback is lacking, so too are PPI contributors’ means of knowing whether, how or where their contributions may have been useful and so limit opportunities to identify, improve and enhance PPI in on‐going studies. Absence of feedback can also lead to reduced motivation among PPI contributors to be involved in future projects.^12^ The authors of a recent study confirm the continuing absence of individual feedback, concluding that “…feedback was seen as an important driver of impact improvement and motivation to stay involved in research”^16^ ^(p.525)^. Thus despite PPI guidance mentioning feedback,^11^ the extent and variation of feedback from researchers to members of the public are not well‐evidenced. This paper reports on the findings of a study which explored feedback from researchers to PPI contributors in health research to address this gap in the literature. Importantly, it was members of the public who initially identified this study idea as addressing a vital area for research.

The aim of this study was to determine variation in types, extent, importance of, and satisfaction with feedback given by researchers to PPI contributors and identify barriers to PPI feedback. The term ‘Patient and Public Involvement’ is defined by NIHR INVOLVE as “research being carried out ‘with’ or ‘by’ members of the public, rather than ‘to,’ ‘about’ or ‘for’ them”^11^ ^(p.6)^. Members of the public include patients, potential patients, carers as well as people from organizations that represent people who use services^11^ and terms such as public contributor, service user and lay representative^17^ are used. This study uses the term “PPI contributor.”

## 2 METHODS

### 2.1 Study design

An explanatory mixed method sequential study design was utilized with a questionnaire survey followed by semi‐structured interviews. The sequential design enabled the findings from the questionnaire to inform the interview schedule,^18^ and the data collection methods were connected as the questionnaire identified the interview participants.^19^ The questionnaire was anonymous but after completion participants were asked if they were willing to be interviewed and if so, they were asked to provide contact details. PPI contributors were involved in all stages of the research process, (see Table 1) and research meetings and PPI decisions were documented.^20^ The aims of PPI were to initiate and steer the direction of the research, keep the study relevant, collect and interpret data and act as links to other PPI organizations.

**Table 1:** Examples of patient and public involvement in the study

Dates	PPI Research activity	Who
January ‐December 2015	Application for funding. Design of protocol and study design	Subgroup of PPI Regional Working Group formed to look at PPI impact & feedback (6 PPI groups were represented: PPI leads, PPI contributors and researchers)
March 2016	Project starts; design of materials (data collection tools, questionnaire, interview schedule, information leaflets) for ethics submission	PPI Research Group (6 PPI leads, 9 PPI contributors) and 3 researchers. Face‐to‐face meetings, teleconference, email/text/post
July 2016	Data analysis of survey	PPI leads, PPI contributors meeting
Nov 2016	Discussion of findings	PPI leads, PPI contributors and researchers
July ‐ Dec 2016	Interviews, data analysis (read interview transcripts and identify/discuss themes)	1 PPI contributor carried out 2 interviews 4 PPI contributors from PPI Research Group involved in data analysis
November 2017	Dissemination at a national conference	A PPI contributor, PPI lead and researcher co‐present

### 2.2 Setting and participants

Participants were purposively recruited via six PPI groups (four NHS and two University groups) who are members of the PPI Regional Working Group (RWG). The PPI groups ranged in size from 15 PPI contributors to 70 with variation between the structures and methods of working; four groups described themselves as public panels, one a patient panel, one condition specific and all had a research focus. All groups had a PPI lead who circulated research documents to PPI contributors (by email or in paper format) and invited comments via the same method of distribution as well as face‐to‐face meetings. Communication between the researchers and PPI contributors was largely channelled through the PPI lead, especially when projects were being developed.

All 227 PPI contributors from the six PPI groups’ databases (aged 16 or over) and 316 researchers, who had used the PPI groups in the last 18 months, were sent an invitation to take part.

### 2.3 Data collection

The PPI leads (researchers did not have access to PPI groups’ database) distributed the online questionnaire (Survey Monkey) during May 2016 with a reminder email sent three weeks later (paper versions were also available). The questionnaire (which had been piloted) had both closed and open questions and the topics included frequency, mode, type, timing, importance, satisfaction of feedback, what constitutes good feedback and barriers to feedback provision: the qualitative interviews covered similar topics (see Supporting Information). All interviews were carried out via the telephone apart from three participants who were interviewed face to face. All interviews were recorded using digital audio equipment and written consent was obtained from all participants. Interviews were carried out by two researchers (EM/HW) and one PPI contributor (DM) who all had previous interviewing experience. The interviews were transcribed verbatim.

### 2.4 The sample

The characteristics of the questionnaire participants are listed in Table 2. Fifteen of these questionnaire participants were also interviewed; six researchers and a subsample of nine of 32 PPI contributors, who gave their contact details, were purposively selected using a maximum variation sampling approach (PPI group, demographic characteristics (age, gender), PPI and feedback experience).

**Table 2:** Characteristics of questionnaire participants

	PPI Contributors n = 68		Researchers n = 39	
	n	%	n	%
Gender				
Male	23	34	12	31
Female	43	63	27	69
Indeterminate	2	3	0	0
Total	68	100	39	100
Age group				
16‐25	2	3	0	0
26‐55	15	22	28	72
56‐65	17	25	9	23
66‐75	21	31	2	5
76+	13	19	0	0
Total	68	100	39	100
Employment				
Employed	16	24	‐	‐
Clinician	‐	‐	8	21
Chief investigator	‐	‐	19	49
Researcher	‐	‐	19	49
Unemployed	1	2	‐	‐
Student	2	3	1	3
Retired	45	66	‐	‐
Carer	3	4	‐	‐
Other (more than one reply possible; percentages add up to more than 100)	6^a^	9	5^b^	13
Length of experience of PPI				
0‐3years	31	46	17	44
3‐5years	12	18	5	13
5‐10years	11	16	11	28
10years +	13	19	6	15
Total	68	100	39	100
Missing	1	2	‐	‐

a The PPI “other” category included health reasons for not working (n = 4), voluntary work or PPI.

b The researcher “other” category included Principal investigator, research nurse, sponsor, lecturer.

### 2.5 Data analysis

The quantitative survey data were transferred (via Excel) to SPSS (version 23.0) and paper copies were manually entered. Descriptive statistics were obtained to summarize the attributes of the participants and provide an overview of the variables. Participants were compared on frequency of feedback, type of feedback and satisfaction with feedback using the chi‐squared test, Fisher's exact test, the Mann‐Whitney *U* test and Kruskal‐Wallis one‐way analysis of variance. Comparisons were adjusted for length of involvement and number of previous studies as PPI contributors had suggested that these factors might affect satisfaction. Initial identification of themes from the qualitative sections of the survey was carried out at a face‐to‐face meeting with the wider group of PPI contributors and PPI leads (Table 1). The qualitative open questions from the questionnaire and the interview data were entered into NVIVO (Version 11). The two sources of qualitative data were integrated through merging, and the interview themes were initially mapped onto the survey themes.^19^ The qualitative data analysis took an analytical approach which was guided by the principles of the constant comparative method.^21^ Three PPI contributors (DM, NR and GR) read two interview transcripts each, two researchers (EM/HW) read all the interviews and four PPI contributors (DM, NR, GR and PM) met to discuss interview themes. Analysis was carried out both within and between themes and care was taken to avoid decontextualization by referring back to the original data and context. Saturation of data was achieved when no new themes emerged. The integration of the quantitative and qualitative data occurred at the interpretation level with both findings being presented together on a thematic basis.^19^

### 2.6 Ethics

The study received approval from the Proportionate Review Subcommittee of the North West ‐ Liverpool Central Research Ethics Committee (REC 16/NW/0245; IRAS 203158) in April 2016. Approval was also obtained from the Research and Development offices for four National Health Service (NHS) Trusts where four of the PPI groups were based.

## 3 FINDINGS

A total of 107 participants completed questionnaires; 68 PPI contributors (10 completed on paper) and 39 researchers (Table 2). This was a response rate of 30% for PPI contributors and 12% for researchers. The quantitative data from the survey questionnaire are presented alongside qualitative quotations from PPI contributors (PPICo) and researchers (Res) from the questionnaire (Quest:) and interview (Int:) data according to the main themes identified.

Patient and public involvement varied considerably from 6 months to over 10 years’ experience for both researchers and PPI contributors. The questionnaire findings revealed that PPI contributors and researchers had experience throughout all stages of the research cycle from priority setting through to dissemination. Involvement in research design was the most common PPI activity, with 75% of PPI contributors involved at the design stage and 95% of researchers stating that they had involved PPI contributors at this stage. PPI contributors and researchers identified research design as being the most useful stage for PPI.

The main findings are presented in five parts with subsections; firstly, the meanings of “feedback” are presented, secondly, the extent and variation of feedback (frequency, mode, type), thirdly, the importance of feedback, fourthly, satisfaction of feedback and lastly, the barriers to providing feedback.

### 3.1 Definitions of feedback

It was a deliberate decision by the research team not to provide the questionnaire and interview participants with a specific definition of “Feedback,” as it was felt important that an exploratory approach was adopted and participants were able to provide their own interpretation. The answers are grouped into three broad themes: an acknowledgement, impact on research and study success and progress.

#### 3.1.1 Feedback: An acknowledgement

The importance of receiving an acknowledgement from researchers was a recurrent theme from both questionnaire and interview participants. For some PPI contributors, acknowledgement was all they wanted “just an acknowledgement…yes that's all” (Int: PPICo14). PPI contributors wanted to know that their comments had been received as some were unsure if their comments had reached the researcher, especially when sent via their PPI Lead:

Acknowledgement at least is common courtesy as not everyone has confidence in the robustness of email routes e.g. the vagaries and delays encountered by nhs.net. email addresses!! (Int: PPICo15)

This theme of feedback as good manners or politeness was also expressed by researchers;

They are acting as experts/team members…disrespectful not to acknowledge this and send thanks and feedback (Quest: Res17)

#### 3.1.2 Feedback: Impact on research

Patient and public involvement contributors also wanted to know if their comments were useful and made a difference to the research, for example if their comments had been implemented, had their input changed anything, had comments been considered or had the comments confirmed researchers’ ideas. One PPI contributor defined good feedback as;

Information on how PPI has influenced any change in design, content, participants, etc. (Quest: PPICo44)

Some PPI contributors and researchers agreed that (where possible) good feedback should be specific and provide detail regarding what changes had been made or not and the reasons why, as these two quotations demonstrate:

I think feedback should be specific, as opposed to simply saying their feedback was helpful. Explain how it was helpful, what changes were made as a result of their comments etc. (Quest: Res04)

Probably a synthetic but full account of how, where and why (and why not) their input was useful, showing how it changed a protocol or an idea (Quest: Res39)

However, some PPI contributors recognized that individual feedback may not be realistic.

One PPI contributor who had been a co‐applicant outlined their experience of good feedback;

showing whether things have been actioned or not and that was all done via tracked changes and so again you could see quite tangibly whether what you've said has been taken into account (Int: PPICo48)

These quotations refer to the fact that PPI comments are not always used: however, it was still felt to be important for researchers to explain and feedback. One PPI contributor interviewee said they were unsure if they wanted to hear negative feedback.

#### 3.1.3 Feedback: Study success and progress

A third feedback theme was feedback on study application success (in terms of funding or ethics approval) and also feedback about the on‐going progress of the study (ie. research stage, papers published, outcomes of the research and summary of project). One PPI contributor felt that feedback *gives a clear understanding of progress (or not) of the research project” (Quest: PPICo34)*.

### 3.2 Frequency, mode and type of feedback

One of the aims of the questionnaire survey was to find out the extent to which PPI contributors received feedback on their comments. The findings showed that nearly one in five (19%) of PPI contributors never received feedback whilst one‐tenth (11%) of researchers said they never gave feedback (Table 3). Sixty‐five percent of PPI contributors and 45% of researchers said they sometimes received (and gave) feedback.

**Table 3:** Frequency of feedback (Questionnaire Results) Q: Do you generally receive/give feedback on your/PPI comments?

	PPI Contributors		Researchers	
	n	%	n	%
Never	12	19	4	11
Sometimes	40	65	17	45
Always	10	16	17	45
Total	62	100	38	100^a^

Missing: PPI contributors = 6; Researchers = 1.

a Percentages may not total 100 due to rounding.

One PPI contributor who had been involved with 13 studies and had not received any feedback on their last five projects wrote

I have to say that even though I think this is important I rarely receive any feedback from comments I have made (Quest: PPICo13)

However, this participant's experience was unusual; generally a range of feedback experiences developed as involvement continued. PPI contributors who reported receiving feedback ‘sometimes’ had been involved in more studies (median number: ‘always’ 2.0, ‘sometimes’ 13.0, ‘never’ 2.5; *P* = 0.003, Kruskal‐Wallis ANOVA). A corresponding pattern emerged as researchers gained experience. Those who reported giving feedback ‘sometimes’ had been involved in more studies (median number: ‘always’ 2.0, ‘sometimes’ 5.5, ‘never’ 1.0; *P* = 0.010, Kruskal‐Wallis ANOVA).

Of those participants who indicated they had received feedback, over two‐thirds (67%) of PPI contributors received feedback direct from researchers and 22% from a PPI coordinator or lead (the remainder reported a mixture). The most common method reported by PPI contributors for receiving feedback was by email (74%) followed by face to face (44%). Half of researchers said they fed back directly to PPI contributors. The role of the PPI leads appears important in facilitating feedback, in terms of raising the issue and encouraging researchers to feedback. The following quotations demonstrate that the PPI leads help to facilitate initial interactions, liaise, remind and “*badger the researchers*” (Int: PPIRep15).

because you can't have feedback if you haven't got for example a lead PPI person on the research team, somebody needs to take ownership, somebody needs to be responsible for providing the feedback (Int: PPICo15)

that [PPI lead] post in itself makes all the difference and obviously in making the feedback cycle, like she's part of that cycle of making sure that the feedback… (Int: PPICo51)

The range of feedback (Table 4) was also explored in the questionnaire with researchers and PPI contributors asked “what was the most common sort of feedback they received or gave?”. Researchers said they mainly gave feedback to PPI contributors that their comments were useful and secondly, gave details about the changes made and had a dialogue (communication back and forth). PPI contributors reported receiving a wider range of types of feedback.

**Table 4:** Most common types of feedback (Questionnaire Results) Q: In general, which sort of feedback is the most common?

	PPI Contributors		Researchers	
	n	%	n	%
I do not hear anything/I generally do not give feedback	7	15	0	0
I hear through the PPI lead or I give feedback via the PPI lead	8	17	2	6
They acknowledge my comments have been received	8	17	2	6
They let me know my comments were useful	3	6	12	33
They let me know my comments led to changes (no details)	2	4	0	0
They let me know my comments led to changes (detailed)	8	17	8	22
They let me know why they did not use my comments	0	0	0	0
They let me know they would like more comments	0	0	0	0
We have a dialogue back and forth	4	8	8	22
Other (comments included feedback very variable, it depends)	5	10	4	11
More than one answer	3	6	0	0
Total	48	100	36	100

Missing or not applicable: PPI contributors = 20 Researchers = 3.

There were some recurring themes in the qualitative data on the manner and characteristics of good feedback; participants suggested a dialogue, discussion, face to face, the tone of feedback (honest, frank, integrity) and timely;

a polite and honest conversation on how useful or not their comments were (Quest: Res02)

Given that PPI contributors had different interpretations of what feedback meant and that different PPI activities (commenting on documents compared with being on an advisory group) require different types of feedback, it was generally recognized that there was not one way to give feedback, as commented by a number of participants;

Unfortunately there are as many good ways to provide “good” feedback as there are PPI representatives! (Quest: PPICo17)

good feedback varies and depends on a number of variables including experience of the PPI representative, what has been requested and the nature and quantity of the feedback; at least it should provide acknowledgement and thanks for receipt and, at most, it should describe how the [PPI] feedback has been used (Int: PPICo105)

The importance of researchers and PPI contributors discussing their expectations at the beginning of the research process was highlighted.

different PPI members have different expectations, many will tell us if they want to know more, others do not appear to want more than what the other members of the team receive (Int: Res03)

### 3.3 Importance of feedback

The questionnaire revealed that for the majority of PPI contributors receiving feedback was very or quite important (82%) to them with a similar proportion of researchers stating that giving feedback was very or quite important (87%). The qualitative data from the interviews and the open questions in the questionnaire provided more insight into the reasons why feedback was considered important. PPI contributors rated feedback as important for several reasons, including appreciation, value, respect, motivation, building confidence and learning and development. Feedback was also seen as giving reassurance to PPI contributors that they are not wasting their time and as motivation for further involvement. Quotations from PPI contributors and researchers illustrate each of these respectively.

Firstly, PPI contributors and researchers agreed that feedback was a sign of appreciation, value and being respectful;

They've given their time to provide input it's respectful to give your feedback (Quest: Res28)

One PPI contributor felt it was a *“social obligation”* to provide feedback

It is a moral, and social obligation, as well as a point of respect for the commitment of the time given by those being asked to give their time, experience, to value their contribution – by providing them with feedback (Quest: PPICo100)

Understanding the relevance/accuracy of feedback assists in my ongoing training. If I spend (my valuable!) time on providing feedback, I'd like to feel that my input is valued but more importantly, know whether my comments were taken on board, and if not, why not (Quest: PPICo16)

Secondly, feedback was viewed as an important motivator for further, future PPI;

Feedback enables PPI members to feel valued, encouraging further involvement in PPI work. Increases PPI confidence and understanding about the research process (Quest: PPICo15)

Thirdly, PPI contributors saw feedback as helping to increase confidence and were also a way of learning how to improve their input;

Feedback helps to ensure that my input is useful, relevant and clearly presented. As a lay person I find that constructive and honest feedback increases my confidence and makes me feel that my input is worthwhile (Quest: PPIRep35)

I feel that it is valuable to be involved in a co‐production with researchers, and receiving this feedback teaches me as well as informing researchers (Quest: PPIRep50)

One PPI contributor reported that without feedback they were forced to guess what the researcher wanted.

Researchers agreed that there should be acknowledgement of time, making PPI contributors feel valued and motivated but did not stress the learning and development theme as much. However, a minority of researchers did not think feedback was important, expected or helpful:

Our patient representatives didn't request any feedback on their input and we weren't told that this was expected so I don't know if they feel it is important (Quest: Res20)

Their comments are their opinion so I am not sure what feedback would be helpful (Quest: Res25)

I can recognise that people working as PPI members of the research team deserve to know about how they influenced the study and indeed if anyone paid attention to what they said, but I don't do this for other members of the team, I don't tell the statistician that their input made a difference for example, I would tell them if I chose to ignore advice on sampling, depending on who it is and how well I knew them I might hesitate to be that explicit with a PPI member of the team (Quest: Res03)

In the last quote, the researcher justifies not giving feedback by treating all members of the research team equally. This theme was explored further in the interviews with a general feeling for a need for equity so that PPI contributors were enabled to be involved to their best ability.

Researchers also mentioned the importance of building relationships as part of giving something back to PPI contributors, with feedback described as a reciprocal relationship;

And I think the more you give to them the more they give back to you….I mean we have to encourage that goodwill by being polite and, you know, inclusive and giving feedback. We have to give, so I do think that's really important (Int: Res18)

Emotionally I think, for me, the impression I get it's [feedback] emotionally important as well as rationally or practically important (Int: PPIRep24)

Reciprocity was seen as both parties gaining benefit from the relationship. Interviewees described the PPI process and feedback using directional terminology, such as PPI being “a two‐way communication” (Int: Res18) or ideally a continual feedback “loop” (Quest: PPICo30), counteracting a one‐way dynamic.

Interview participants discussed feedback as remaining important over time even for those PPI contributors who had engaged in PPI for an extended period. This was highlighted for individuals experiencing fluctuating health, who had to take time out and also for those involved in multiple PPI activities. However, interviewees suggested that reasons to give and receive feedback may change over the life of a project or as PPI contributors gain experience. It was suggested that there may be a temporal aspect to feedback, initially for reassurance and boosting confidence and later relating to PPI contributors’ impact on the research. Those PPI contributors who are providing their personal lived experience as their basis of involvement may require different types of feedback.

### 3.4 Satisfaction with feedback

Of those PPI contributors who had received feedback, one‐quarter (25%) were generally very satisfied with the feedback and two‐fifths (42%) were fairly satisfied. However, some PPI contributors were either fairly unsatisfied (15%) or very unsatisfied (4%). Researchers were also asked if they were satisfied with the feedback they gave, as shown by Table 5.

**Table 5:** Satisfaction with PPI feedback (Questionnaire Results) Q: In general, how satisfied are you with the feedback you receive/give?

	PPI Contributors		Researchers	
	n	%	n	%
Very satisfied	12	25	5	15
Fairly satisfied	20	42	19	56
Neither	7	15	8	24
Fairly unsatisfied	7	15	2	6
Very unsatisfied	2	4	0	0
Total	48	100^a^	34	100^a^

Missing or not applicable: PPI contributors = 20 Researchers = 5.

a Percentages may not total 100 due to rounding.

Satisfaction was analysed as very or fairly satisfied versus other responses. The length of involvement and the number of previous studies participants had been involved in were not associated with satisfaction for either the PPI contributors or the researchers. Analyses were therefore not adjusted for these variables. Table 6 shows that there was a positive association between satisfaction and timely feedback (*P* = 0.007) for PPI contributors. There were also associations between satisfaction and the type of feedback; comments had been useful (*P* = 0.015) and comments had led to changes—details given (*P* = 0.008). For researchers, there was a marginal association between giving feedback as dialogue and satisfaction (*P* = 0.054). Therefore, PPI roles, such as being a co‐researcher, which involves dialogue and face‐to‐face contact, may potentially be more satisfactory in terms of feedback. The qualitative data support these findings with PPI contributors dissatisfied with the lack of feedback, demonstrating lack of respect and value (as outlined earlier).

**Table 6:** Feedback characteristics and satisfaction of PPI contributors with feedback

	Yes	No	Difference (%)	*P*‐value
Timely feedback	26/33 (79%)	2/8 (25%)	54	0.007
Informed that comments				
Have been received	14/19 (74%)	18/28 (64%)	9	0.542
Were useful	19/22 (86%)	13/25 (52%)	34	0.015
Had led to changes (no details)	16/19 (84%)	16/28 (57%)	27	0.063
Had led to changes (details)	15/16 (94%)	17/31 (55%)	39	0.008
Told more comments would have been liked	6/6 (100%)	26/41 (63%)	37	0.157
Dialogue with researcher	10/12 (83%)	22/35 (63%)	20	0.288
Received feedback by				
e‐mail	23/33 (70%)	8/12 (67%)	3	1.000
Telephone	4/6 (67%)	27/39 (69%)	−3	1.000
Face to face	17/21 (81%)	14/24 (58%)	23	0.121
Letter/paper	6/6 (100%)	25/39 (64%)	36	0.156

Of those 37 PPI contributors who had been involved in at least five studies, there was a positive association between receiving feedback in three of more out of the last five studies and general satisfaction (94% vs 0%, *P* < 0.001). This shows perhaps not surprisingly that the recent experience of feedback is particularly important. There was a corresponding finding for researchers. For those 14 researchers who had been involved in at least five studies, there was a positive association between giving feedback for each of the last five studies and general satisfaction (86% vs 17%, *P* = 0.029).

### 3.5 Barriers to feedback

Many participants, both researchers and PPI contributors, identified similar perceived reasons for some researchers not providing feedback: particularly citing time (too busy, hectic project activities, timescale pressures) and budget constraints. The research environment was described as being very fluid with researchers moving between projects or working on several projects at once, which again was considered to hinder feeding back. Such reasons have also been cited elsewhere as reasons for not undertaking PPI.^1^ More specific reasons for not providing feedback and not using PPI comments were word limits, too many comments and PPI contributors’ lack of understanding of the research process. There may be a time delay between PPI contributors’ commenting and the impact of those comments being seen. Participants suggested researchers may not prioritize PPI and feedback and as discussed earlier, researchers may not even know PPI contributors were expecting feedback or that feedback was an expected part of the process. Lastly, it was suggested researchers may not have the necessary sensitive communication skills or are reluctant to deal with sometimes complex relationships.

## 4 DISCUSSION

The study had PPI contributors involved throughout, including initiation, and to our knowledge, this is the first mixed methods study, internationally, to focus on the role of feedback from researchers to PPI contributors in health research. Our research does resonate with previous work on the importance of understanding people's values and motivations for undertaking PPI and specifically what they expect as part of their experience, including getting feedback.^1^, ^22^ A key finding is that not all researchers provide feedback or provide satisfactory feedback all of the time and this is viewed negatively by many PPI contributors. There is also a disparity between PPI expectations for feedback and the priority it is given by researchers. Our study found different meanings of the term PPI feedback which included acknowledgement, impact and study success and progress. Both PPI contributors and researchers saw an acknowledgement of the contribution as a minimum requirement for feedback and more detailed comments were preferred to enable PPI contributors to feel valued and motivated. However, unsurprisingly, it seems that there is no generic way in which to provide PPI feedback.

The findings of this study suggest that improving the frequency and quality of feedback provided to PPI contributors could bring a range of important benefits; firstly, by enhancing PPI contributors’ experience (feeling valued, appreciation of time commitment) and motivation for future involvement; secondly, to PPI contributors’ learning and development (getting feedback on how to improve their comments to researchers); and thirdly, improving research practice with researchers needing to reflect on the PPI, routinely documenting PPI impact and so strengthening the PPI evidence base. Thus identifying ways to improve PPI feedback communication has both a methodological and moral rationale. However, this study has identified that not all researchers recognize the learning element and there have been on‐going debates about whether PPI contributors should be trained or ‘professionalised’ or remain ‘lay’.^23^, ^24^ Our findings demonstrate how PPI provides a learning opportunity for both PPI contributors and researchers, as discussed previously by Staley.^25^ Our research suggests that additional training may be required for researchers to help them to communicate feedback in a sensitive, constructive way. This research has highlighted that some researchers/PPI contributors may not feel comfortable giving or receiving negative feedback and much depends on the nature of the feedback, how it is given and the context in which it is transacted. The challenges for researchers in providing feedback should be recognized.

Comprehensive international, national and local guidance for PPI is available.^11^, ^26^ Guidance in the United Kingdom reflects different meanings of the term PPI feedback and over time there has been a shift in emphasis from feedback as a “thank you” to feedback as “evaluation of impact”.^27^ The shift is reflective of a more general move towards measuring PPI impact.^28^, ^29^ INVOLVE guidance mentions feedback in terms of project results; “feedback results to all those you consulted and collaborated with as well as participants”.^11^ Our study revealed that although PPI guidance recommends researchers provide feedback to PPI contributors, there are few routine PPI feedback structures in place. The importance of feedback is not restricted to health research and also applies to patients/services users involved in health services^30^, ^31^ and study participants.^32^

Many recent studies and toolkits have highlighted the importance of PPI contributor and researcher relationships^1^, ^33^ and findings from this study confirm its significance and complexity. However, the three‐way relationship between a researcher, PPI lead and PPI contributor is less well‐known or discussed. The importance of this relationship emerged as a finding from this research. It is clear that PPI leads were seen as positive facilitators for enabling successful PPI and feedback can act as an important mediator between researchers and PPI contributors, especially for document review, one‐off or new involvement. However, their presence may also create some distance between the two and may result in researchers not being aware of the needs or expectations of the PPI contributors. The PPI lead adds complexity to the dyad of researcher‐PPI contributor communication and relationship. In those cases where PPI contributors are not attached to a PPI group (and PPI lead), it will be necessary for someone in the research team to take responsibility for feedback.

It is acknowledged that this small‐scale study has limitations. Although statistically significant associations with satisfaction were identified, due to the low numbers completing the questionnaire, it was not possible to conduct multivariable analyses making the drawing of conclusions difficult. In addition, bias may be an issue due to the low response rate and those who filled in the survey and took part in the interviews may be atypical of the wider population. The distribution of respondents across the six PPI groups may also be a limitation.

## 5 CONCLUSION

In the pressured world of short‐term contract‐based health research, PPI and feedback are not always seen as a priority despite PPI now being a policy imperative and prerequisite for health research.^34^ However, PPI feedback should be an integral part of the wider research process to be designed and costed, and then systematically reviewed so as to maximize mutual benefits and continuing learning from the PPI contribution. This research has demonstrated that PPI feedback can be viewed as a key mechanism for successful, embedded PPI.^35^ Feedback is a demonstration of PPI maturing and becoming an intertwined part of the PPI process. Feedback can enable PPI contributors to feel valued, motivated and learn, and for researchers, feedback offers the opportunity to reflect on PPI impact thus providing the potential to improve the PPI evidence base and research practice.

## CONFLICT OF INTEREST

No conflict of interests.
