# Supplementary material for: Reciprocal relationships and the importance of feedback in patient and public involvement: A mixed methods study
Source: Health Expect. 2018 Apr 14;21(5):899–908. doi: 10.1111/hex.12684 (PMC6186542; doi:10.1111/hex.12684)
Supplement: Supplementary file 1 [file HEX-21-899-s001.docx]

| Completing the Feedback Cycle: Survey of PPI Representatives 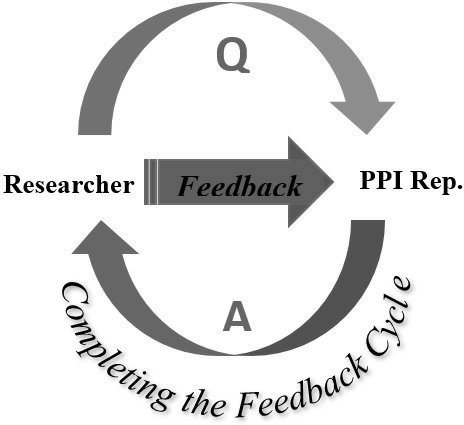 |  |
| --- | --- |
| *Questions 1-4 will help us describe our survey respondents* |  |
| 1. What gender do you consider yourself to be?  Male 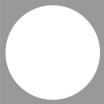 Female 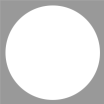 Indeterminate 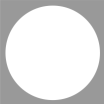 2. Which age group do you fit within?  16-25 years old 26-35 years old 36-45 years old 46-55 years old 56-65 years old 66-75 years old 76 and over 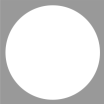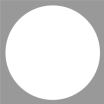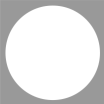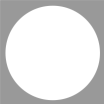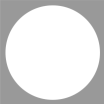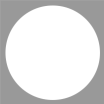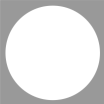 3. What is your marital status?  Single 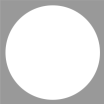 Married 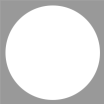 Living together 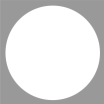 Widowed 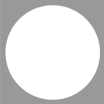 Divorced / separated 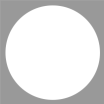 In a relationships but none of the above 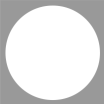 I don't wish to say 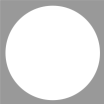 |  |

4. What is your employment status? You may select more than one.

Employed (PT/FT) Unemployed Student

Retired

Carer

Other (please specify)

5. Which PPI group are you attached to? You may tick more than one (Groups were named).

Group 1

Group 2

Group 3

Group 4

Group 5

Group 6

Other Group

Other (please specify)

6. Approximately, how long have you been doing this role as Patient and Public involvement?

Under 6 months


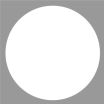


More than 6 months - 1 year More than 1 year - 2 years More than 2 years - 3 years More than 3 years - 4 years More than 4 years - 5 years More than 5 years - 10 years Over 10 years


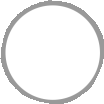

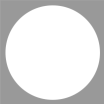

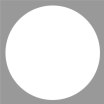

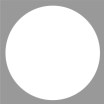

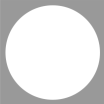

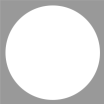

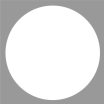

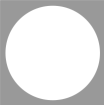


Other (please specify)

7. What stages of the research process have you been involved in? You may tick more than one.

Priority setting (coming up with a research question/helping decide what research to do)

Design of research (input into design and commenting on protocols, patient information sheets) Management of research (Advisory Boards / Steering Committees)

Undertaking research (carrying out interviews, data analysis) Dissemination of findings (talking/writing about the results) Co-researcher

Co-applicant

Other (please specify)

8. At what stage of the research cycle do you think PPI is most useful?

Priority setting (coming up with a research question/helping to decide what research to do)


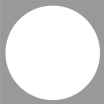


Design of research (input into design and commenting on protocols, patient information sheets) Management of research (Advisory Boards / Steering Committees)


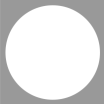

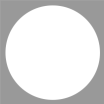


Undertaking research (carrying out interviews, data analysis) Dissemination of findings (talking/writing about the results) Co-researcher


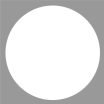

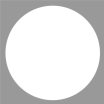

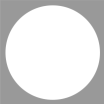


Co-applicant


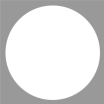


Other (please specify)


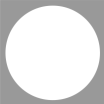


9. How important is PPI in research to you?

Very important


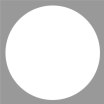


Quite important


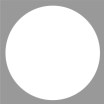


Not especially important


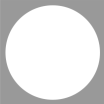


Not at all important


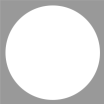


No opinion


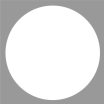


It depends


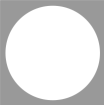


Other (please specify)


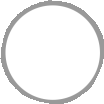


10. Why do you feel this level of importance about PPI in research?


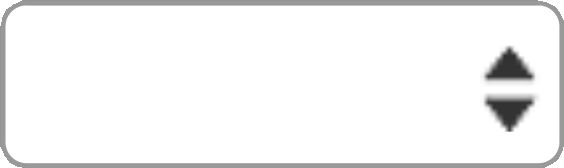

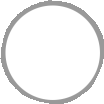

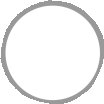

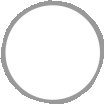

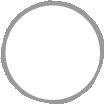

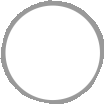

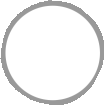

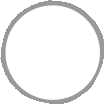

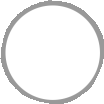

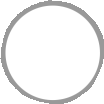

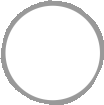

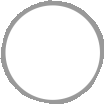


11. How important is it to you that researchers give feedback (on your comments) to you personally?

Very important

Quite important

Not especially important

Not at all important

No opinion

It depends

Other (please specify)

12. Why do you place this level of importance on receiving feedback?

13. Can you estimate how many research studies you have been involved with (giving PPI advice not as a study participant)? If it is a high figure, please just estimate.

14. In the last five research studies you have been involved with how many have you received feedback from the researchers about your comments?

15. Who have you received feedback from?

A member of the research team

The PPI co-ordinator / Lead (the person who runs the PPI group) Other

for 'other ' or 'it depends' answers please explain

16. Do you generally receive feedback on your comments?

Always


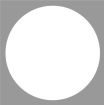


Sometimes


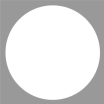


Never - if never please skip to Q.24 at the end of the survey to leave your comments


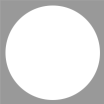


Comments

17. Do you feel that the feedback you receive is timely?

Yes


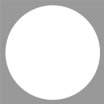


Not applicable


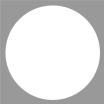


No (please state how the period of time you would have liked to receive comments in)


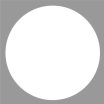


18. In general, when you make comments on research documents or provide other contributions, what sort of feedback do you receive either from the researchers or through your PPI Lead? You may tick more than one.

I do not hear anything

I do not hear from the researcher directly but through the PPI co-ordinator/lead (person who runs the PPI group) They acknowledge my comments have been received

They let me know my comments were useful

They let me know my comments led to changes/modifications (not detailed)

They let me know my comments led to changes/modifications and these are detailed (i.e. track changes on an information leaflet, sent new copy of document)

They let me know why they did not use my comments

They let me know they would like more comments

We have a dialogue/conversation (back and forth) about my comments

Not applicable

Other (please specify)

19. In general, when you make comments on research documents or provide other contributions, which sort of feedback is the most common?


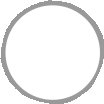

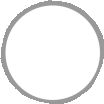

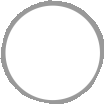

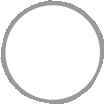

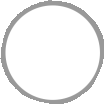

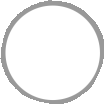

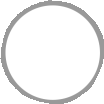

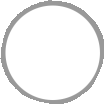

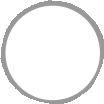

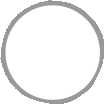

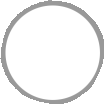


I do not hear anything

I do not hear from the researcher directly but through the PPI co-ordinator/lead (person who runs the PPI group) They acknowledge my comments have been received

They let me know my comments were useful

They let me know my comments led to changes/modifications (not detailed)

They let me know my comments led to changes/modifications and these are detailed (i.e. track changes on an information leaflet, sent new copy of document)

They let me know why they did not use my comments

They let me know they would like more comments

We have a dialogue/conversation (back and forth) about my comments

Not applicable

Other (please specify)

20. How do researchers let you know their feedback? You may tick more than one.

Email Telephone Face to face Letter / paper Not applicable

Other (please specify)

21. How would you like to receive feedback?

22. In your last project how satisfied were you with the feedback you received?

Very satisfied Fairly satisfied Neither


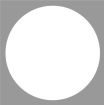

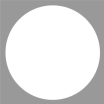

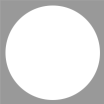


Fairly unsatisfied Very unsatisfied Not applicable


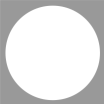

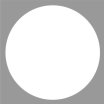

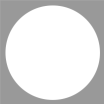


23. In general, how satisfied are you with the feedback you receive?

Very satisfied Fairly satisfied Neither


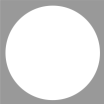

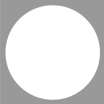

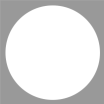


Fairly unsatisfied Very unsatisfied Not applicable


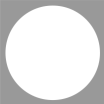

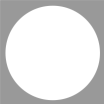

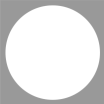


24. Why do you think researchers do not provide feedback?

25. What do you think is good feedback to PPI representatives?

26. How can PPI feedback be improved?

27. Please add any other comments:

28. If you would like to be invited to take part in an interview, please give your contact details (email address or phone number) so that we can contact you to provide you with more information. You are under no obligation to take part. Your details will be kept securely.

“PLEASE DO NOT COPY OR REPRODUCE THIS QUESTIONNAIRE WITHOUT PERMISSION FROM THE AUTHORS”
