# Supplementary material for: Reciprocal relationships and the importance of feedback in patient and public involvement: A mixed methods study
Source: Health Expect. 2018 Apr 14;21(5):899–908. doi: 10.1111/hex.12684 (PMC6186542; doi:10.1111/hex.12684)
Supplement: Supplementary file 2 [file HEX-21-899-s002.docx]

**PPI Feedback Cycle:**

**Interview Questions**

**PPI Representatives**

- Why do you do PPI?
- Is getting feedback (from researchers) on your comments important to you?
- What sort of feedback do you want on your comments?
- Are you satisfied with the feedback you get?
- Have you ever discussed feedback with a researcher?
- How do you think feedback could be improved?
- Do you think your need for feedback will change over time?
- When do you think you should receive feedback? (time)
- What challenges are there for researchers to provide good feedback?

**Researchers**

- Is it usually you or a member of your research team that provides feedback?
- Why do you involve PPI representatives?
- Do you think feedback is important to PPI representatives, if so why?
- What sort of feedback do you give?
- Do you think the PPI representatives you work with are satisfied with the feedback you give?
- Have you ever discussed feedback with them?
- How do you think feedback could be improved?
- Has your PPI feedback changed over time?
- When do you think you should give feedback? (time)
- What challenges do you have in giving feedback to PPI representatives?
- Do you think the feedback you provide could form part of your interim final reports (PPI reporting/Key performance indicators (KPI))?

**“*PLEASE DO NOT COPY OR REPRODUCE THIS INTERVIEW SCHEDULE WITHOUT PERMISSION FROM THE AUTHORS”***
